# Evaluation of psychometric properties and differential item functioning of 8-item Child Perceptions Questionnaires using item response theory

**DOI:** 10.1186/s12889-015-2133-3

**Published:** 2015-08-19

**Authors:** David TW Yau, May CM Wong, KF Lam, Colman McGrath

**Affiliations:** Dental Public Health, Faculty of Dentistry, The University of Hong Kong, 34 Hospital Road, Hong Kong SAR, China; Department of Statistics and Actuarial Science, Faculty of Science, The University of Hong Kong, Hong Kong SAR, China

## Abstract

**Background:**

Four-factor structure of the two 8-item short forms of Child Perceptions Questionnaire CPQ_11–14_ (RSF:8 and ISF:8) has been confirmed. However, the sum scores are typically reported in practice as a proxy of Oral health-related Quality of Life (OHRQoL), which implied a unidimensional structure. This study first assessed the unidimensionality of 8-item short forms of CPQ_11–14_. Item response theory (IRT) was employed to offer an alternative and complementary approach of validation and to overcome the limitations of classical test theory assumptions.

**Methods:**

A random sample of 649 12-year-old school children in Hong Kong was analyzed. Unidimensionality of the scale was tested by confirmatory factor analysis (CFA), principle component analysis (PCA) and local dependency (LD) statistic. Graded response model was fitted to the data. Contribution of each item to the scale was assessed by item information function (IIF). Reliability of the scale was assessed by test information function (TIF). Differential item functioning (DIF) across gender was identified by Wald test and expected score functions.

**Results:**

Both CPQ_11–14_ RSF:8 and ISF:8 did not deviate much from the unidimensionality assumption. Results from CFA indicated acceptable fit of the one-factor model. PCA indicated that the first principle component explained >30 % of the total variation with high factor loadings for both RSF:8 and ISF:8. Almost all LD statistic <10 indicated the absence of local dependency. Flat and low IIFs were observed in the oral symptoms items suggesting little contribution of information to the scale and item removal caused little practical impact. Comparing the TIFs, RSF:8 showed slightly better information than ISF:8. In addition to oral symptoms items, the item “Concerned with what other people think” demonstrated a uniform DIF (*p* < 0.001). The expected score functions were not much different between boys and girls.

**Conclusions:**

Items related to oral symptoms were not informative to OHRQoL and deletion of these items is suggested. The impact of DIF across gender on the overall score was minimal. CPQ_11–14_ RSF:8 performed slightly better than ISF:8 in measurement precision. The 6-item short forms suggested by IRT validation should be further investigated to ensure their robustness, responsiveness and discriminative performance.

## Background

Assessing the impact of oral diseases/conditions on children’s quality of life had been neglected until Jokovic et al. [1] raised the awareness. Child Perceptions Questionnaire (CPQ_11–14_) was developed in Toronto as a pioneer instrument on children’s oral health-related quality of life (OHRQoL) consisting of 4 domains, namely oral symptoms, functional limitation, emotional well-being and social well-being. The original 37-item CPQ_11–14_ was then shortened into 16- and 8-item CPQ_11–14_ by item-impact method (Item-impact Short Forms: ISF:16/ ISF:8) and regression method (Regression Short Forms: RSF:16/ RSF:8) [2]. Furthermore, it was translated into different languages and validated including Portuguese [3], German [4], Arabic [5] and Chinese [6]. Traditional validation procedures have been extensively applied on CPQ_11–14_ for both 37 items and short forms, such as internal consistency, test-retest reliability and criterion, convergent and discriminant validity [2–9]. Further, structural equation modelling and factor analysis also confirmed the hypothesized factor structure of CPQ_11–14_ RSF:8 and ISF:8 [10]. Currently, there are just a few applications of CPQ_11–14_ short forms in epidemiological and clinical studies [11, 12]. However, these short forms should be promoted by considering the potential benefits such as reducing the respondents’ burden and non-response, saving time and cost [8].

Item response theory (IRT) offers an alternative and complementary approach to validate and explore the psychometric properties of instruments. It has potential to solve some problems incurred by the classical test theory, such as: (i) items are assumed to be weighted equally; (ii) the test properties depend on the sample; (iii) only one constant reliability estimate of the scale; (iv) the presumption of interval scale to ordered response categories. Moreover, the IRT approach can also serve as a mean to investigate item bias by differential item functioning (DIF) analysis.

Despite confirmation of the 4-factor structure [10], reporting of the total score remains a common practice which implicitly assumed a one-dimensional nature of the scale. Discrepancy arises in the practical use of sum score of CPQ_11–14_ as a measure of OHRQoL and the theoretical factor structure. In view of this, the present study intended to test empirically to what extent OHRQoL can be treated as a one dimensional construct.

Although both short forms were proven valid and reliable in classical test theory analysis, practitioners may remain arbitrary in deciding which short forms to be used. This study used the IRT approach to evaluate the item properties of CPQ_11–14_ ISF:8 and RSF:8 that cannot be uncovered by classical test and compare whether the two short forms performed similarly.

Furthermore, the questionnaire should work the same way in any respondent [13]. Measurement equivalence of CPQ_11–14_ across different language versions has been assessed using DIF technique [14]. However, research concerning DIF across gender of CPQ_11–14_ is scant. Boys and girls (at the age of 12) may perceive the items differently and this results in biased scores. In this study, DIF across gender and its potential impacts were also assessed.

## Methods

### Sample

The participants were secondary school students recruited for an observation survey to study the association between dental caries and adiposity status [15]. In brief, the primary sampling unit was secondary school and the sampling frame was the list of Hong Kong local secondary schools. About 10 % of local secondary schools were randomly drawn from the 18 districts in Hong Kong. Within each secondary school, all students from S1 and S2 (equivalent to US grades 6 and 7) who were born in April 1997 and May 1997 were invited to this study. Data were collected from January to April 2010 and all participants were 12 year-old. Written consent was obtained from parents or caregiver of each participant. Students were asked to provide their assent. The study protocol was approved by the Institutional Review Board of the University of Hong Kong/Hospital Authority Hong Kong West Cluster (WU09-435).

### Measures

Participants were asked to complete a questionnaire which consisted of both CPQ_11–14_ RSF:8 and ISF:8 items, questions concerning their global self-health-ratings, dietary habits, oral hygiene behaviors and demographics backgrounds. Participants completed the questionnaires in a self-administered mode. Clinical oral examination and anthropometric assessment were also conducted. Only CPQ_11–14_ RSF:8 and CPQ_11–14_ ISF:8 data collected through the questionnaire were used in the current study. For each question in the CPQ_11–14_ participants were asked “In the past 3 months, how often have you … (had/been)…because of your teeth/mouth?”. The five Likert response categories were: ‘Never’ = 0; ‘Once/twice’ = 1; ‘Sometimes’ = 2; ‘Often’ = 3; ‘Every day/almost every day’ = 4 [1]. Missing responses were imputed with ‘Never’ = 0 as we presumed children not answering the questions probably had not encountered the situations mentioned in the items. Imputing ‘Never’ = 0 was previously used to handle questionnaires with a “Don’t know” option [13]. Questionnaires with more than 2 missing items will be discarded from this analysis.

### Statistical analysis

The mathematical foundation of IRT lies on relating the items’ characteristics in an instrument to the probability of choosing a particular response option taken into account the respondents’ levels of latent construct (which is OHRQoL in this study) [16].

Item response analysis assumes the latent construct (OHRQoL) is adequately represented by the items. Another requirement to warrant substantive interpretation of the result is local dependency. Local dependency implies that items residuals do not correlate to each other. Although in reality data sets rarely comply fully to underlying assumptions [17], various techniques allow us to explore the degree to which the assumptions are met. For the assessment of dimensionality, principal component analysis (PCA) and confirmatory factor analysis (CFA) were carried out. In PCA, evidence supporting dominance of a general factor was in particular interest. Indicators include factor loadings of the items [18], the percentage of variance explained by the first principal component (PC) and ratio of eigenvalue of first PC to that of the second [16]. In CFA, the model fit statistics of a one factor model including Chi-square test, root mean square error approximation (RMSEA), normative fit index (NFI), comparative fit index (CFI), goodness of fit index (GFI) and standardized root mean square residual (RMSR) were investigated. NFI, CFI and GFI values should be greater than 0.9; while RMSR and RMSEA should be less than 0.08 for adequate fit [19]. Local dependency statistic (LD) tests for the correlation of every pair of items residuals [20] at which LD greater than 10 indicated the presence of local dependency [21].

The CPQ_11–14_ data were fitted by Samejima’s graded response model (GRM) [22]. The GRM was formulated as:

$$ \log \left(\frac{{P^{+}}_{j,k}}{1-{P^{+}}_{j,k}}\right)={\mathrm{a}}_{\mathrm{j}}\left(\uptheta \hbox{-} {\mathrm{b}}_{j,k}\right), $$

where P^+^_*j,k*_’s is the probability of choosing the *k + 1*^th^ or higher response options for the *j*^th^ item; *a*_*j*_’s represent the item discriminatory parameters and *b*_*j,k*_’s are the item threshold parameters for the *k*^th^ response option in the *j*^th^ item; *θ* is the person’s OHRQoL. S-χ^2^ test, adjusted for the model-dependent observed proportion, was used for assessing the goodness of fit of each item, i.e., discrepancy of model’s prediction for each item and the observed data [23]. Further, the overall goodness of fit of the GRM model could be assessed by RMSEA as a supplement in the case of large sample size [24]*.*

Since higher score of CPQ_11–14_ represents poorer OHRQoL and a standard normal distribution was assigned to the OHRQoL spectrum, respondents’ OHRQoL were mapped to a scale of −3 to 3. Respondents with average OHRQoL were mapped to zero on the scale; those with poorer than average OHRQoL were mapped on the positive range of the scale, while those with better than average OHRQoL were mapped on the negative range of the scale.

The threshold parameters (*b*_*j,k*_) and discriminatory parameters (*a*_*j*_) were the primary outcomes of the item response model. The threshold parameter (*b*_*j,k*_) represented the OHRQoL level that respondents would equally prefer the *k + 1*^th^ response option or above to other options in the *j*^th^ item. For example, b_*j,1*_ represents the OHRQoL level which a person would equally prefer the 2^nd^ or above options (“Once/ twice” = 1 to “Every day/ almost every day” = 4) to the 1^st^ option (“Never” = 0); b_*j,2*_ represents the OHRQoL level which a person would equally prefer the 3^rd^ or above options (“Sometimes” = 2 to “Every day/ almost every day” = 4) to the 1^st^ or 2^nd^ response option (“Never” = 0 or “Once/twice” = 1). The discriminatory parameters (*a*_*j*_) indicated the relative importance or contribution of the *j*^th^ item in discriminating different OHRQoL, i.e., whether a change in OHRQoL could lead to adequate change in the probabilities of answering different response options in the *j*^th^ item. For items with low discriminatory power, people of different OHRQoL level would choose the response options with similar chances.

Item response theory offers a mean to identify biased items through the investigation of DIF. Non uniform DIF and uniform DIF occurs respectively when discriminatory parameters (*a*_*j*_) and threshold parameters (*b*_*j,k*_) vary across sub-populations. It was tested whether boys and girls may view items differently by investigating DIF across gender. Items parameter (*a*_*j*_ and *b*_*j,k*_) that differ significantly across gender are considered biased items. Wald test was used to detect DIF [25, 26]. Since too few respondents chose ‘Everyday/almost every day’ in some items, response options ‘Often’ and ‘Everyday/almost every day’ were combined in DIF analysis. To assess the effect size of DIF, the expected score for boys and girls were calculated [27].

Test information function (TIF) and item information function (IIF) are powerful tools for describing and comparing instruments [16]. Test information reflects how precisely the latent construct is estimated. Item information provides insight on contribution of each item to the precision of the scale. This is the analogy to the concept of reliability in classical test theory. In this study, the IIF and TIF of the two short form versions of CPQ_11–14_ were examined and compared.

IBM SPSS 20 was used to perform PCA and generate other descriptive statistics. CFA was performed by LISREL8.80 [28]. IRTPRO (Item Response Theory for Patient-Reported Outcomes) student version was used throughout this study for item response analysis [21].

## Results

### Participants

A random sample of 668 students aged 12 completed the questionnaire. 19 respondents with missing responses for more than 2 items were excluded. Out of 649 respondents, 319 (49.2 %) were male. The mean scores for CPQ_11–14_ RSF:8 and CPQ_11–14_ ISF:8 across gender are shown in Table 1.

**Table 1:** CPQ_11–14_ RSF:8 and ISF:8 scores by gender

	RSF:8	ISF:8
	Means (SD)	Means (SD)
Boys	6.9 (3.9)	7.6 (3.8)
Girls	7.1 (3.7)	7.3 (3.5)
Overall	7.0 (3.8)	7.4 (3.7)

### Dimensionality

**Fig. 1:**
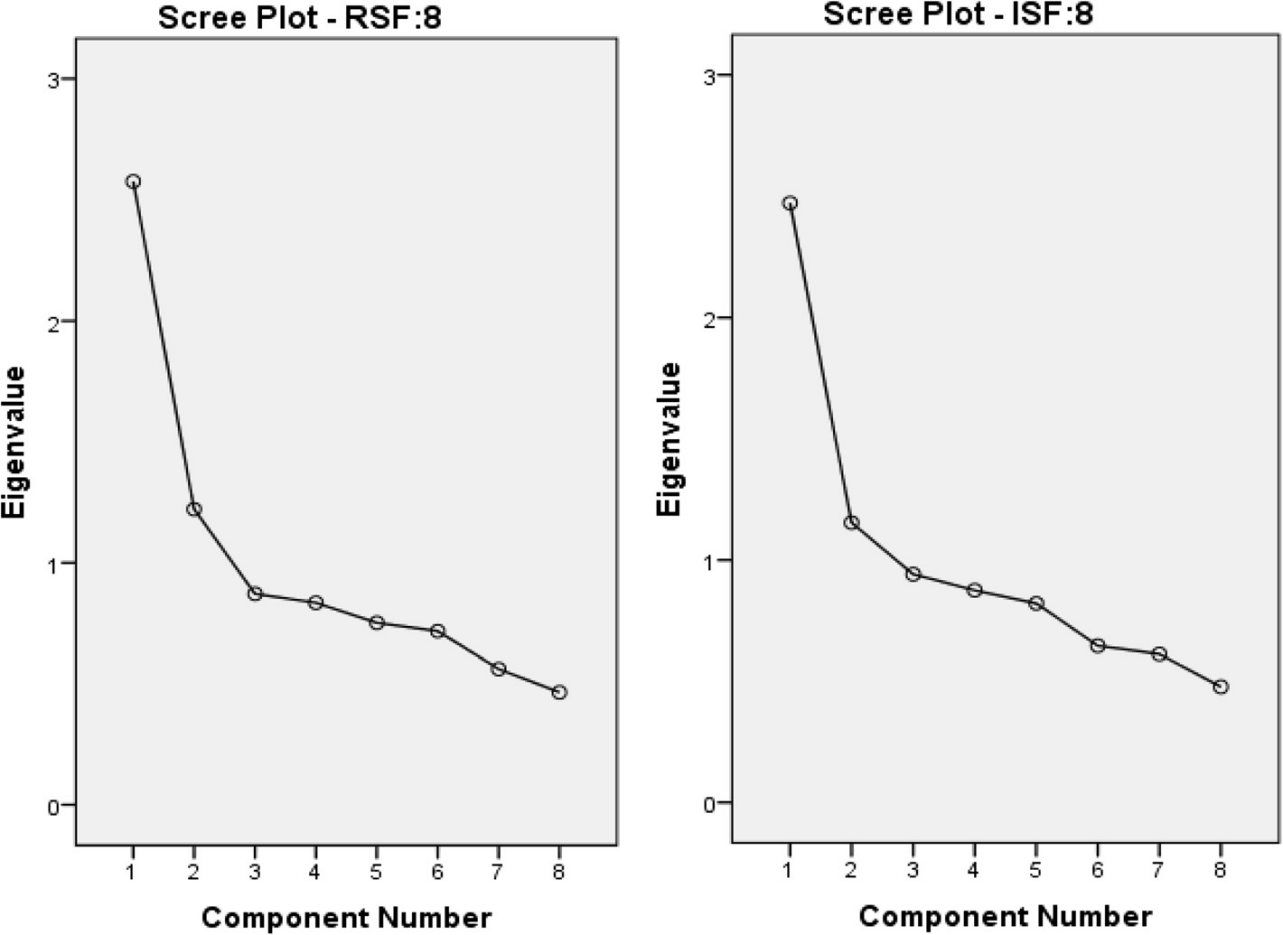
Scree plots of RSF:8 and ISF:8

Summary results of PCA and CFA assessing the unidimensionality hypothesis are shown in Table 2. In PCA, percentage of variance explained by the first principal component (PC) for both RSF:8 and ISF:8 were >30 %. The ratios of first eigenvalue to that of the second were 2.11 and 2.22 for RSF:8 and ISF:8 respectively. Scree plots (Fig. 1) suggested the dominance of the first general factor. For the first PC, 7 out of 8 factor loadings in RSF:8 and all factor loadings in ISF:8 > 0.33. The item in RSF8 with relatively low factor loading (0.27) was “Mouth sores”. In CFA, RMSR, GFI, CFI and NFI supports the one-factor model of RSF:8. GFI and RMSR supports the one-factor model of ISF:8 whereas weak support was obtained from other fit statistics.

**Table 2:** Fit index for unidimensionality assumption

	RSF:8	ISF:8
PCA		
% of variance explained by first PC	32.2 %	30.9 %
Ratio of first PC to second PC	2.11	2.22
Number of first PC factor loading >0.33	7 out of 8	8 out of 8
CFA		
RMSEA	0.088	0.102
NFI	0.90	0.84
CFI	0.91	0.85
p-value (Chi-square test)	<0.001	<0.001
GFI	0.96	0.94
Standardized RMSR	0.041	0.043

### Calibration and item fit

Graded response model (GRM) was calibrated. RMSEA = 0.03 showed that data fit the GRM well. S-χ^2^ test for item fit is shown in Table 3. The item: “Irritable/ Frustrated” in ISF:8 had the *p*-value <0.01.

**Table 3:** Item parameter estimates and fit statistics of GRM

	a	b_1_	b_2_	b_3_	b_4_	S-*χ* ^2^	df	p
RSF:8								
Oral symptoms								
1. Mouth sores	0.45	−2.54	0.08	8.01	12.18	61.4	60	0.426
2. Bad breath	0.53	−3.01	−0.68	4.31	7.29	75.8	77	0.517
Functional limitations								
3. Trouble sleeping	1.17	0.00	1.58	3.16	4.41	68.7	59	0.181
4. Difficult to say any words	1.11	0.81	2.65	4.87	6.33	46.2	45	0.423
Emotional well-being								
5. Concerned with other people think	1.40	−0.34	0.90	2.68	3.76	61.9	59	0.372
6. Upset	1.98	0.07	1.24	2.77	3.81	51.4	45	0.236
Social well-being								
7. Argued with other children or your family	1.65	0.06	1.24	2.95	4.00	62.9	47	0.061
8. Teased/called names by other children	1.34	0.17	1.55	2.83	3.69	57.9	57	0.445
ISF:8								
Oral symptoms								
1. Bad breath	0.53	−3.01	−0.68	4.31	7.29	75.8	77	0.517
2. Food caught between/in teeth	0.63	−5.39	−2.78	2.48	7.7	62.4	61	0.424
Functional limitations								
3. Difficult to bite or chew food like apples, corn on the cob or steak	0.96	0.14	2.29	4.77	6.47	56.5	53	0.344
4. Difficult to drink or eat hot or cold foods	0.94	0.35	1.97	4.09	5.1	72.6	59	0.110
Emotional well-being								
5. Irritable/frustrated	1.84	−0.13	1.00	2.34	3.35	89.1	58	0.005
6. Upset	1.98	0.07	1.24	2.77	3.81	51.4	45	0.236
Social well-being								
7. Avoided smiling/laughing when around other children	1.58	0.66	1.87	2.97	3.9	59.3	49	0.149
8. Asked questions about your teeth, lips, jaws or mouth by other children	0.95	0.18	2.3	4.91	6.07	56.5	51	0.276

Estimated threshold parameters (*b*_*j,k*_’s) of GRM are presented in Table 3. In both RSF:8 and ISF:8, items concerning oral symptoms had lower threshold parameters compared to others i.e., individuals were prone to answer higher response options in items concerning oral symptoms compared to other items.

For items other than those concerning oral symptoms, the threshold parameters b_j,1_ were close to 0, i.e., respondents with better than average OHRQoL would most likely answer “Never” to these items. This pattern of threshold parameter was an indication of floor effect. In all items, threshold parameters b_j,3_ were at least 2.3, i.e., when assuming a standard normal distribution to population OHRQoL, approximately only the worst 1 % individuals would prefer “Often” or “Everyday/almost every day” to preceding response options.

Interpretations of threshold parameters *b*_*j,k*_ were confounded to discriminatory parameters *a*_*j*_. Oral symptom items in both RSF:8 and ISF:8 had small discriminatory parameters. Small discriminatory parameters imply that probabilities of responding to each option were not different regardless of the respondents’ OHRQoL. Almost all the LD statistics <10 indicated a weak local dependency.

### Reliability

**Fig. 2:**
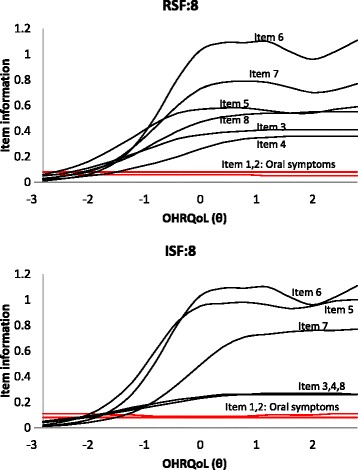
Item Information functions

Plots of IIF of each item in RSF:8 and ISF:8 against the OHRQoL (θ) were shown in Fig. 2. The item information curves of items concerning oral symptoms were particularly low in the entire OHRQoL scale. These suggested oral symptoms hardly added value to the precision of OHRQoL. Therefore these items were identified as non-informative items and this echoed the low discriminatory power of these items. Items contributing most information were all under the domain of emotional and social well-being.

**Fig. 3:**
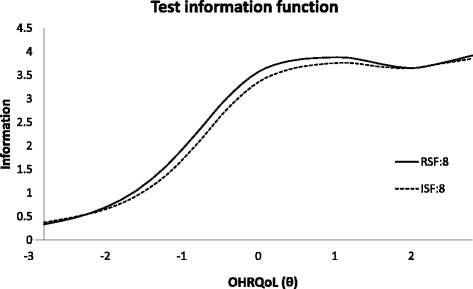
Test information function

Fig. 3 illustrates that TIFs of both RSF:8 and ISF:8 were higher at the right end of the scale (worse OHRQoL) which indicated that more precise OHRQoL was estimated for people with worse OHRQoL. TIF also allowed us to compare the 2 short versions of CPQ_11–14._ The TIF of RSF:8 was slightly higher in most part of the OHRQoL scale, i.e., RSF:8 provides a more precise estimate for OHRQoL than ISF:8.

### Differential item functioning (DIF)

**Fig. 4:**
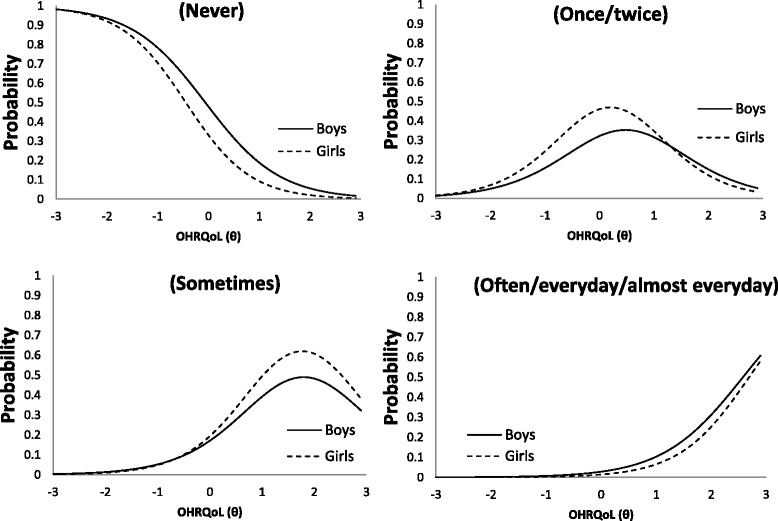
Item characteristic curve of the item “Concerned with what other people think” for male and female

**Fig. 5:**
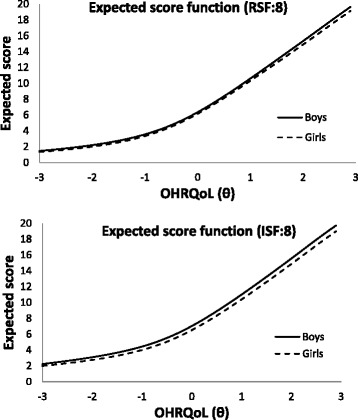
Expected score for male and female

Table 4 presents items with DIF across gender. Non-uniform DIF was not found but three items exhibited uniform DIF across gender: “Bad breath” (in both RSF:8 and ISF:8), “Food caught between/ in teeth” (in ISF:8), and “Concerned with what other people think” (in RSF:8). For item “Bad breath”, with the same level of OHRQoL, boys are less likely to give a response of “Never” and “Once or twice” than girls. For the item “Food caught between teeth”, girls were more likely to answer “Once or twice a day” but less likely for “Often/everyday/almost every day”. For the item “Concerned with what other people think”, girls were prone to answer “Sometimes” and “Once or twice” (Fig. 4). DIF was not considered a practical problem because the differences in expected scores were small (<1-point along the whole OHRQoL scale) (Fig. 5).

**Table 4:** Items with DIF across boys and girls

Item	Gender	a	b_1_	b_2_	b_3_
Bad breath	Boys	0.63	−3.01	−1.05	3.14
(in both RSF:8 and ISF:8)	Girls	0.52	−2.50	−0.04	5.38
		(*χ* ^2^ = 0.4, df = 1, *p* = 0.540)	(*χ* ^2^ = 19.0, df = 3, *p* < 0.001)		
Food caught between/in teeth	Boys	0.65	−5.00	−2.95	1.99
(in ISF:8)	Girls	0.67	−5.16	−2.27	2.93
		(*χ* ^2^ < 0.1, df = 1, *p* = 0.943)	(*χ* ^2^ = 12.6, df = 3, *p* = 0.006)		
Concerned with other people think	Boys	1.37	−0.06	1.02	2.58
(in RSF:8)	Girls	1.57	−0.45	0.85	2.69
		(*χ* ^2^ = 1.5, df = 1, *p* = 0.483)	(*χ* ^2^ = 11.5, df = 3, *p* = 0.009)

### Removal of symptom related items

**Fig. 6:**
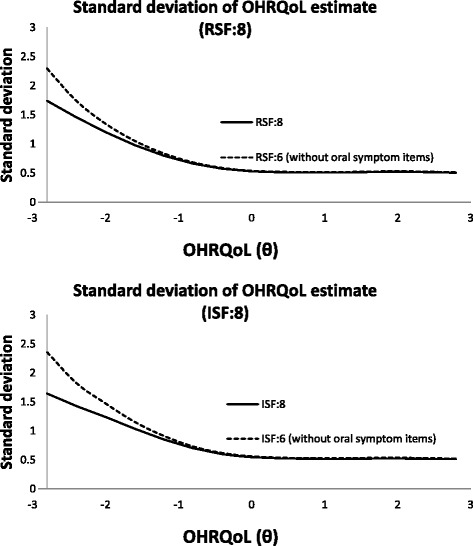
Standard deviation of OHRQoL estimate with and without oral symptoms items

**Fig. 7:**
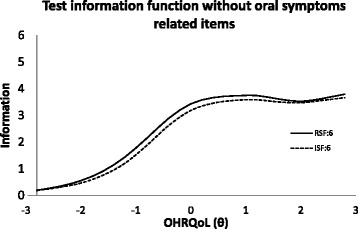
Test information function after removal of items related to oral symptoms

Since items concerning oral symptoms were not informative to OHRQoL and subjected to DIF, removal of items was considered, resulting in RSF:6 and ISF:6. The impact of removal of symptom related items is shown in Fig. 6, which plotted respectively the information function of CPQ_11–14_ with and without items concerning oral symptoms. Negligible impact was made on the standard deviation of OHRQoL estimates on majority of the OHRQoL scale. However, the standard error of OHRQoL increased obviously for people with good OHRQoL, i.e., for people with good OHRQoL (better than average by about 1 standard deviation), their estimated OHRQoL would be less precise. This is still considered acceptable because reducing the 2 oral symptom items does not undermine its ability to distinguish poor OHRQoL people. Upon removal of the oral symptom items, the TIF of RSF:6 was also slightly higher than that of ISF:6 in most of the OHRQoL scale (Fig. 7).

## Discussion

The purpose of this study was to evaluate the psychometric properties of the 8-item short forms CPQ_11–14_ by IRT model. Special attention has been paid to the investigation of the unidimensionality assumption of the IRT because CPQ_11–14_ was originally designed with 4 subdomains under the umbrella of OHRQoL but usual practice of using sum score implies unidimensionality. It is important to strike a balance of simplicity and completeness of model [17]. While different approaches to assess dimensionality exists, no clear cutoff is provided [29]. In view of this, various approaches were adopted to explore the degree of unidimensionality of RSF:8 and ISF:8. Despite mixed evidence of unidimensionality, one-dimensional IRT was used because: (i) principle of parsimony using simple model to explain reality [30]; (ii) when IRT was performed on each subdomains, there would only be 2 items in each subdomain which arguably would affect reliability and content-validity [17].

It was observed that in both RSF:8 and ISF:8 the estimated discriminant parameters were low and the information was flat in items concerning oral symptoms: bad breath, mouth sore and food caught in between teeth. This result concurs with a study on the factor structure on these two questionnaires where factor loadings on symptoms items were particularly low [10]. It implies that oral symptoms contribute little to OHRQoL. However, this is in contrast to previous suggestion of oral symptoms as a subdomain of OHRQoL [31, 32]. Two possible explanations of this phenomenon are suggested as follow. First, respondents were only asked to report the frequency of oral symptoms but not severity. The prevalence of oral symptoms was higher than that of other items; however, the severity could vary. The majority of healthy individuals are likely to have mild degree of oral symptoms. Second, OHRQoL is a psychological concept whereas symptoms are objective physical aspects. It is the impact of oral symptoms, rather than symptom itself, that is important. Studies have identified that some patients with quite severe chronic diseases have reported good quality of life [33]. Another study (on cancer patients) also showed that the effect of symptoms on quality of life was more significantly affected by patients’ resilience than symptoms [34]. Health psychologists recognized that characteristics of individuals including optimism and resilience could be associated with OHRQoL [35, 36]. The present study raises the need for further study on the moderation effect of psychological assets on the relationship between symptoms and OHRQoL. Future research on the possibility of psychological intervention as an alternative to improve OHRQoL is warranted.

The present study confirmed that the symptom related items in both CPQ_11–14_ RSF:8 and ISF:8 added little value in measuring OHRQoL, especially in identifying people with poor OHRQoL. Since CPQ_11–14_ targeted to identify people with poor OHRQoL, the removal of 2 oral symptoms items post little practical impact. However, a limitation of this study is the lack of data for a thorough investigation of the relationship of oral symptoms to OHRQoL. This study was originally aimed only to study the psychometric properties of 2 short forms of CPQ_11–14_. Therefore, only items belonging to these short forms were used in these questionnaires. Although the symptom related items in both 8-item short forms of CPQ_11–14_ was confirmed not useful, valid conclusion about the relationship between oral symptoms and OHRQoL for 12-year old children cannot be drawn. Future research should be performed to explain this interesting phenomenon and understand the underlying relationship between oral symptoms and OHRQoL for people of different age group.

Gender DIF analysis identified 3 uniform DIF items – 2 of them were under the domain of oral symptoms. Regarding “Concerned what other people think”, it was found that girls were prone to respond to more frequent response options as shown in Fig. 4. This could possibly be explained by the fact that girls at the age of 12 are more sensitive to their appearance and impression. Three approaches were proposed to handle DIF items: (i) ignore the DIF, (ii) form separate scale for different groups and (iii) delete or modify the item [27]. Fig. 5 shows that the difference in the expected scores between groups was not greater than 1 (out of the possible range of 0–32) and rather uniform across the scale. This implied that the DIF was of little practical significance in spite of the statistical significance. Another purpose in this study was to compare the performances of RSF:8 and ISF:8 which were well validated in previous researches by traditional methods [2, 10]. In this study, evaluation criteria were based on the differential item functioning and test information function. Although some items parameters across gender were detected to be differed significantly, they were of little practical impact.

The sampling method of this study entails a representative sample of Hong Kong lower secondary school children. Therefore, the psychometric properties discussed can comfortably be applied locally. Extrapolation of the psychometric properties to other countries has to be done with caution. When considering DIF, understanding of each item across gender may depend on the social norm or environment which vary across countries. Researchers should use item response theory to investigate the item contribution in other countries to confirm whether the items’ contribution of CPQ_11–14_ is consistent across countries.

## Conclusions

This study illustrated the use of item response theory in reporting and comparing the metric properties of 8-item short forms CPQ_11–14_. The unidimensional structure to infer OHRQoL is acceptable. Items concerning oral symptoms contributed little to the OHRQoL scale. This evidence does not support the use of frequency of oral symptoms in OHRQoL measurement and deletion of oral symptoms related items from RSF:8 and ISF:8 is suggested. Both 8-items short forms can measure people with worse OHRQoL more precisely. CPQ_11–14_ RSF:8 performed slightly better than ISF:8 in terms of measurement precision regardless of the deletion of oral symptom related items. Although items with differential item function across gender were identified, its impact on the overall score was minimal. The removal of oral symptoms items resulting in 6-item short forms suggested by IRT validation should be further investigated to ensure their performance to be robust, discriminative and responsive.

## Abbreviations

CFA: Confirmatory factor analysis
CFI: Comparative fit index
CPQ_11–14_: Child Perceptions Questionnaire
DIF: Differential item functioning
GFI: Goodness of fit index
GRM: Graded response model
IIF: Item information function
IRT: Item response theory
ISF:6: 6-item Short form CPQ_11–14_ obtained by removal of 2 symptom related items from ISF:8
ISF:8/ ISF:16: 8-/16-item Short form CPQ_11–14_ obtained by item impact method
LD: Local dependency
NFI: Normative fit index
OHRQoL: Oral health-related quality of life
PC: Principle component
PCA: Principle component analysis
RMSEA: Root mean square error approximation
RMSR: Standardized root mean square residual
RSF:6: 6-item Short form CPQ_11–14_ obtained by removal of 2 symptom related items from RSF:8
RSF:8 / RSF:16: 8-/16-item Short form CPQ_11–14_ obtained by regression method
TIF: Test information function
